# Using Communication to Modulate Neural Synchronization in Teams

**DOI:** 10.3389/fnhum.2020.00332

**Published:** 2020-09-08

**Authors:** Terri A. Dunbar, Jamie C. Gorman

**Affiliations:** Systems Psychology Laboratory, School of Psychology, Georgia Institute of Technology, Atlanta, GA, United States

**Keywords:** team process, team coordination, neural synchronization, team communication, constraints, entropy, cross-level effect

## Abstract

Throughout training and team performance, teams may be assessed based on their communication patterns to identify which behaviors contributed to the team’s performance; however, this process of establishing meaning in communication is burdensome and time consuming despite the low monetary cost. A current topic in team research is developing covert measures, which are easier to analyze in real-time, to identify team processes as they occur during team performance; however, little is known about how overt and covert measures of team process relate to one another. In this study, we investigated the relationship between overt (communication) and covert (neural) measures of team process by manipulating the interaction partner (participant or experimenter) team members worked with and the type of task (decision-making or action-based) teams performed to assess their effects on team neural synchronization (measured as neurodynamic entropy) and communication (measured as both flow and content). The results indicated that the type of task affected how the teams structured their communication but had unpredictable effects on the neural synchronization of the team when averaged across the task session. The interaction partner did not affect team neural synchronization when averaged. However, there were significant relationships when communication and neural processes were examined over time between the neurodynamic entropy and the communication flow time series due to both the type of task and the interaction partner. Specifically, significant relationships across time were observed when participants were interacting with the other participant, during the second task trial, and across different regions of the cortex depending on the type of task being performed. The findings from the time series analyses suggest that factors that are previously known to affect communication (interaction partner and task type) also structure the relationship between team communication and neural synchronization—cross-level effects—but only when examined across time. Future research should consider these factors when developing new conceptualizations of team process measurement for measuring team performance over time.

## Introduction

Cross-level effects, or temporal dependencies across levels of analysis (e.g., physiological, cognitive, and motor), are thought to occur unconsciously and develop naturally as people interact (Gorman et al., 2016). The purpose of the current study is to establish whether cross-level effects exist in teams between the communication and neurophysiological levels of analysis and whether they can be controlled by constraining how teams coordinate. Empirical grounding for this research can be found in studies performed on individuals and teams where cross-level effects occur through the coupling of motor, perceptual, and neural processes during task performance. In individual speech, for example, the speed of speech syllables, the movement of the mouth, and the electrical activity in the auditory cortex all modulate at a frequency between 3 and 8 Hz (Ghazanfar and Schroeder, 2006; Schroeder et al., 2008; Chandrasekaran et al., 2009; Luo et al., 2010). This entrainment across levels of analysis (mouth movements, speech, and auditory neural activity) suggests that these processes (motor, perceptual, and neural) are coupled during the production of speech.

Entrainment also occurs in interpersonal communication where neural synchronization (i.e., temporal coupling due to interacting in a shared medium; Strogatz, 2003) occurs incidentally between people during a conversation. More specifically, the neural activity of the speaker is spatially and temporally correlated with the listener’s neural activity (Stephens et al., 2010; Dikker et al., 2014; Pérez et al., 2017). This neural coupling often occurs at a delay, but it can also be predictive, where the listener’s neural activity anticipates the speaker’s neural activity (Stephens et al., 2010; Kuhlen et al., 2012). Studies using live verbal communication find similar results, especially during face-to-face communication (Jiang et al., 2012; Spiegelhalder et al., 2014). According to Stephens et al. (2010), the neural coupling may serve as a mechanism for how brains coordinate or convey information successfully between individuals. The coupling mechanism is also supported by prior findings that people have similar neural responses when they interpret a narrative the same way (Nguyen et al., 2019). This study will expand on this research by experimentally inducing cross-level effects during live, back-and-forth conversation during more complex team tasks.

In the current study, cross-level effects are experimentally induced in two ways: Type of task and the interaction partner. According to the theory of interactive team cognition, team cognition embodied in team communication is inextricably tied to task context (Cooke et al., 2013). Therefore, we hypothesize that if team communication and neural processes are coupled, then changes in team communication will be concomitant with changes in neural synchronization as a function of task context. In previous research, we found that the type of team task controls the type of team communication pattern that emerges over time (Dunbar and Gorman, 2014). Specifically, dyads performed one of two task types: The Non-combatant Evacuation Operation planning task (NEO; Warner et al., 2003) or the Minecraft visual-spatial coordination task (Dunbar and Gorman, 2014). Dyads that performed the NEO task dynamically organized their communication around planning and cognitive processes whereas dyads that performed the Minecraft task dynamically organized their communication around perceptual information directly available in the environment. Because we have established that these tasks affect the dynamic structure of team communication, we used these two task types to manipulate cross-level effects in the current study.

Although the effects of these two specific tasks on the patterns of neural synchronization in teams are unknown, according to predictions from the dynamical systems theory of team coordination (Gorman, 2014; Gorman et al., 2017b), neural synchronization that occurs within these two tasks should be coupled with the cognitive-behavioral (here, communication) differences between the two tasks. Although it has not been established where neural synchronization should occur in these tasks, we used research on related tasks to inform us where possible indicators of neural synchronization might occur.

Relevant to the NEO planning task, neural synchronization has been observed in submarine crews engaged in planning and visual-spatial coordination using electroencephalogram (EEG) in the 10–12 Hz frequency range using a measure of engagement (Berka et al., 2007) at the sensor sites Fz and POz (Stevens and Galloway, 2016, 2017; Gorman et al., 2016). Higher-level social coordination processes, such as when people are engaged in planning, have also been linked to increased coupling in the mu medial and phi complex rhythms in the alpha band (8–12 Hz) in the FCz and CP4 electrodes of EEG (Tognoli and Kelso, 2015). Abstract communication, such as planning communication, has also been tied to the FC1 and FC2 electrodes in the alpha band (Moreno et al., 2013). This pattern of results suggests that for the NEO task, the Fz, POz, CP4, FCz, FC1, and FC2 electrodes in the alpha frequency range are possible locations to investigate for indicators of team neural synchronization.

Relevant to the Minecraft visual-spatial task, a similar type of task is the map task where people communicate about the shapes and locations of objects on maps. For teams performing the map task, neural synchronization occurred in the 16–17 Hz frequency range of EEG at the sensor sites Fz, C3, and C4 (Stevens and Galloway, 2014, 2016). Action-based communication, which is captured in the perceptual-motor communication of the Minecraft task, has been tied to mu and low beta rhythms in the beta band (13–30 Hz) in the C3, Cz, and C4 electrodes of an EEG (Moreno et al., 2013). These correspond to similar areas associated with motor simulation processes when people observe another person act (Muthukumaraswamy et al., 2004). This pattern of results suggests that for the Minecraft task, the Fz, C3, C4, and Cz electrodes in the beta frequency range are possible locations to investigate for indicators of team neural synchronization.

The overall pattern of the neural activation results suggests that there may be different indicators of neural synchronization depending on the type of task the team is performing. Table 1 summarizes the pattern of research findings on neural synchronization. Based on these findings, we hypothesized that experimentally manipulating the type of team task modulates both communication behavior and neural synchronization, the latter of which may be indicated along with different EEG frequency bands at pre-specified sensor sites such as the Fz, POz, P4, Cz, C3, and C4 electrodes depending on whether the task is more planning-based (NEO) vs. action-based (Minecraft). Because the spatial resolution of EEG is low, we also looked broadly at the frontal, central, and parietal areas for neural synchronization differences between the two tasks.

**Table 1:** Summary of findings on neural synchronization.

Study	Alpha (8–12 Hz)	Beta (13–30 Hz)
Tognoli and Kelso (2015)	**Social Coordination**:
	FCz, CP4	
Stevens and Galloway (2016, 2017)	**Submarine Crews**:	
Gorman et al. (2016)	Fz, POz
Stevens and Galloway (2014, 2016)		**Map Task**:
		Fz, C3, C4
Moreno et al. (2013)	**Abstract**:	**Action**:
	FC1, FC2	Cz, C3, C4
	**Action**:	
	Cz, C3, C4	

We also examined whether neural synchronization between team members depended on whether they were communicating with each other to complete the task vs. communicating with a trained experimenter to complete the task. Our manipulation is based on basic research on interpersonal cognitive and motor processes, such as postural synchronization (Shockley et al., 2003) and visual synchronization (Richardson et al., 2007) during the conversation. In those studies, the researchers provide a control condition to help eliminate the possibility that any observed synchronization is spurious. The control condition consists of the participant communicating with an experimenter to perform the task, whereas the experimental condition consists of the participant communicating with another participant (here, a teammate) to perform the task, with synchronization between participants measured under both conditions. In the experimental condition, synchronization occurs due to the verbal coupling between the participants. However, in the control condition, synchronization between participants does not occur because they are not verbally coupled (i.e., they are coupled with an experimenter). This pattern of results indicates that postural and visual synchronization during conversation is not spurious. We used this same logic to validate spontaneous neural synchronization between verbally coupled participants.

In the current study, participants performed one of two tasks as a dyad, the NEO task, or the Minecraft task (between-subjects manipulation). These tasks were performed twice, once with their teammate and once with an experimenter (within-subjects manipulation). During task performance in all conditions, we continuously measured the EEG of the participants and the communication of both the participants and experimenters. We used these manipulations to test four hypotheses:

*Hypothesis 1*: Dynamic structuring of communication patterns depends on the type of team task being performed. Specifically, we should observe conversations organized around perceptual information for the action-based Minecraft task and around planning and cognitive processes for the planning-based NEO task.

Hypothesis 1 is a manipulation check. The purpose of testing this hypothesis is to ensure that the task type manipulation impacts the dynamics of team communication as expected based on prior research (Dunbar and Gorman, 2014).

*Hypothesis 2*: Neural synchronization between team members occurs when communicating with a teammate but not when communicating with an experimenter.

According to Hypothesis 2, neural synchronization between team members should only occur when the team members are verbally coupled with one another. When team members are communicating with the experimenter they are verbally coupled with the experimenters, and thus synchronization between team members should not occur if verbal coupling with a teammate modulates neural synchronization. The purpose of testing this hypothesis, therefore, is to provide a control condition against which the state of spontaneous synchronization between people during the conversation (i.e., cross-level effects) can be compared.

*Hypothesis 3*: Type of neurophysiological synchronization between teammates depends on the type of team task.

According to Hypothesis 3 and theoretical cross-level effects, Task Type should not only modulate the dynamic structuring of communication patterns but also modulate neural synchronization. Based on prior research, we predicted that indicators of neural synchronization should be found in the alpha band (8–12 Hz) in the Fz, POz, and P4 electrodes for the NEO task, whereas indicators for neural synchronization in the Minecraft task should be found in in the beta band (13–30 Hz) in the Fz, Cz, C3, and C4 electrodes. We also looked more broadly across the frontal, central, and parietal cortical areas for task type differences.

*Hypothesis 4*: Communication and neural activity should be coupled, such that changes in communication patterns should be reflected in the neural activity of teams (or vice versa).

According to Hypothesis 4, if team coordination occurs simultaneously across multiple levels of analysis, then these levels should be temporally related to one another. Moreover, fluctuations in neural synchronization (e.g., periods with high, low, or medium neural synchronization) should temporally lead or lag team communication flow. The weaker version of this hypothesis is that temporal coupling between the neural and communication levels occurs only at the moment (e.g., in the same second), such that no lead-lag relationship exists. To test this hypothesis, we computed lagged temporal cross-correlations between neural synchronization and communication flow time series and analyzed differences in the observed patterns of temporal cross-correlation using chi-square tests of independence.

## Materials and Methods

### Participants and Procedure

Forty-six participants (23 dyads) were recruited from the Georgia Institute of Technology psychology participant pool and were compensated with course credit for completing the study. During the study, 10 dyads’ data were discarded due to equipment failures (microphones that stopped recording and servers crashing), and 1 dyads’ data was discarded due to a participant dropping out of the study early. Data collection continued until 24 participants’ (six dyads per between-subjects condition) complete data sets were available for analyses, which was the desired sample size based on an *a priori* power analysis. The power analysis was conducted using effect sizes (*d* = 2.11 and *d* = 1.89) from a prior study that detected task differences in the communication dynamics of teams using the same tasks as the current study (Dunbar and Gorman, 2014). One team included in the analysis did not have recorded communication data for the experimenter condition, but all neural data for this team was intact. Average participant age was *M* = 19.70 (*SD* = 1.77). The sample was predominantly male, with 28.26% of the participants being female, and the remaining 71.74% male. Eight dyads were mixed gender and four dyads were all male. This study was approved by the Georgia Institute of Technology’s Institutional Review Board and carried out following their recommendations.

Participants were pre-screened using an online recruitment advertisement to avoid possible complications with EEG data collection. Pre-screening included colorblindness, systemic disorders with CNS involvement, neurological disorders, alcohol and drug abuse or dependence, CNS active medications, or psychiatric disorders. Participants were reminded to avoid caffeine, nicotine, and alcohol consumption 24 h before participation. No participants reported knowing each other before the experiment; any recent caffeine, nicotine, or alcohol consumption; alcohol and drug abuse or dependence; CNS active medications; or any systemic disorders with CNS involvement, psychiatric disorders, or neurological disorders. No participants tested as colorblind.

Informed consent was obtained before participation. Dyads were randomly assigned to the Task Type and Trial Order conditions before arriving. Following informed consent, participants were tested for colorblindness because the Minecraft task involves differentiating between different colors of blocks and a red-green colorblind individual would be unable to complete the task; however, participants in both Task Type conditions were given the test to ensure equal treatment. Following the colorblindness test, participants were equipped with the EEG headsets. Before the start of the task, resting baseline EEG power levels were measured for 15 min. Participants then had 15 min to either read through their materials (NEO) or practice the controls of the game (Minecraft), depending on their task condition. The actual task length was 15 min for both tasks, and each task was completed twice. In one task session, participants performed the task together; in the other task session, each participant performed the task with an experimenter. Experimenter task sessions were completed in the same manner as described in the Experimental Design section. Following the second task session, participants filled out the demographics survey and the experimenter debriefed the participants concerning the purposes of the study. Participation lasted 2 h.

### Experimental Design

Participants performed as dyads in one of two task conditions corresponding to a between-subjects variable, Task Type, with two levels, NEO and Minecraft. The interaction served as a within-subjects variable with two levels, where A refers to performing the task with an experimenter and B refers to performing the task with their team member. Participants performed their task twice, either in the AB or BA order, which served to counterbalance the order of trials across teams, and neural synchronization between participants was recorded at each level of Interaction. Finally, a between-subjects variable, Trial Order, indexed the order in which dyads performed the A and B Interaction conditions. This variable was used to check for differential transfer effects.

Two tasks served as the Task Type manipulation for this study: The Non-combatant Evacuation Operation task (NEO; Warner et al., 2003) and the Minecraft task (Dunbar and Gorman, 2014). The NEO task was originally developed by the Navy and was adapted from three participants to two participants for this study. In the NEO task, participants verbally communicate to plan a rescue mission based on a hypothetical military scenario given a limited number of weapons, personnel, and time resources. The team cannot complete the task without verbally communicating because each team member is only given a portion of the information about the resources available for the mission. The team’s goal was to develop a 24 h plan to rescue three Red Cross workers from a church on a fictitious remote island that contains friendly natives and foes. During the task session, the team must plan how they will get the rescuers to the church, how they will evacuate the Red Cross workers, and how they will return to either an Army base or aircraft carrier. To develop their plan, team members communicate about which personnel they will use throughout the mission, plan the specific route the personnel will take on the island to get to the church, how the personnel will extract the Red Cross workers, how they will safely manage the Red Cross workers injuries, and the route the personnel and Red Cross workers will take to return safely. Participants were instructed to type up their planned actions for every hour in 24 h starting at 2:00 AM.

**Figure 1:**
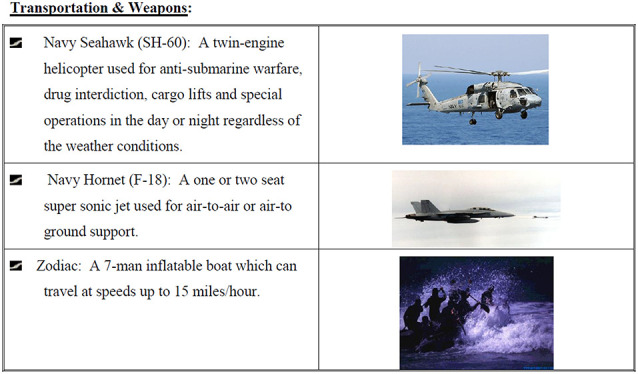
Example of Non-combatant Evacuation Operation (NEO) task materials (Warner et al., 2003). This example contains descriptions of three of the transportation and weapon options available to one of the participants.

Each participant received a file containing general information about the task that included background information about the island, Red Cross workers, rebel forces, military assets, and maps of the island. Participants also received role-specific information containing either weapons expert information or environment/intelligence information that went beyond the general information. The weapons expert information contained information about the various vehicles, weapons, and military personnel that could be used by the participants for their extraction plan. The environmental/intelligence information consisted of weather conditions on the island, information about the water, a more detailed map of the church where the Red Cross workers are located, and information about the island population. For each task iteration, A or B, participants received different information about the island or their resources to minimize a practice or memorization effect. See Figure 1 for examples of information participants received for the NEO task. The participants typed up their plan during each task session in a shared Google Document that both participants accessed through a web browser.

**Figure 2:**
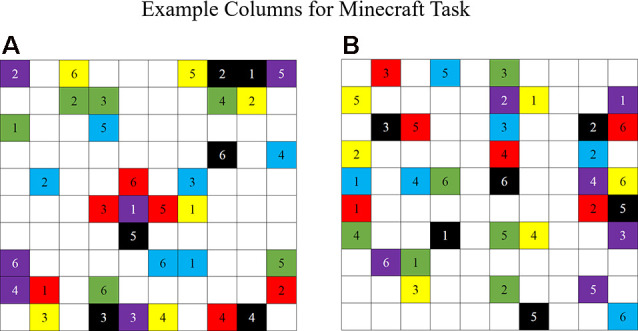
An example set of columns for the Minecraft task (Dunbar and Gorman, 2014). **(A)** Participant 1 set of columns. **(B)** Participant 2 set of columns. Each participant contains a unique set of columns containing six colors: red, green, blue, purple, yellow, and black. Each column varies in height from one to six blocks high.

The second Task Type condition was a one-right answer building task involving building a structure in the game Minecraft (Dunbar and Gorman, 2014). The Minecraft game world is constructed of blocks that players can manipulate by destroying or adding blocks of their own. However, for the task used in this study, participants were given a specific set of blocks to build in the game world and were restricted in the types of blocks they could place in the game. Each participant was given three individual colors of blocks to use in the game. Participant 1 was restricted to red, green, and blue blocks, whereas Participant 2 was restricted to purple, yellow, and black blocks. Also, each participant received 36 columns of blocks to build in the game that required using all six colors of blocks in columns of various heights (Figure 2). Each participant’s set of columns was unique with no overlapping columns between the two sets. Because the participants were only allowed to use three colors of blocks and each participant was given different sets of columns containing all six colors of blocks, the task required participants to verbally communicate with each other to complete the task by placing the correct blocks in the correct sequence to complete the columns. Participants and experimenters (depending on the task iteration) also informed the other participant or experimenter (depending on the task iteration) of blocks that the participant needed them to place in the world to complete their maps. For each task iteration, A or B, participants received a different set of columns to minimize a practice or memorization effect. The team’s goal was to combine both sets of columns into a 10-by-10 square space and verbally communicate the locations, heights, and colors of blocks to be built with each other.

### Apparatus

Participants were seated in separate rooms with the doors closed for the duration of participation, ensuring that participants were unable to see each other and coordinated only through verbal communication. Two experimenters were located in different rooms, adjacent to the participants. Each participant and the experimenters used a computer with dual monitors to complete their tasks. Participants and experimenters communicated with one another *via* microphone using TeamSpeak (TeamSpeak Systems GmbH, 2017) by holding down the shift key in a push-to-talk format.

Dry electrode 20-channel EEG headsets from Cognionics were used for data collection. Cognionics Data Acquisition software (Cognionics, 2018) acquired all neural data from the EEG channels of the two participants. A Cognionics remote trigger was used to synchronize the EEG data streams at every trial. To synchronize the communication data streams with the neural activity data streams, the experimenters stated “We are marking the EEG recording now” into their microphone whenever they pressed the Cognionics remote trigger. The experimenter pressed the trigger when they said the word “now.” The neural activity of the experimenters was not measured because it was not pertinent to the hypotheses of this study.

### Measures

#### Communication Content

We conducted a power-law analysis of the communication content to operationalize the dynamic structuring of the team’s communication in terms of the team’s communication distribution to test Hypothesis 1. To serve as input for this analysis, each dyad’s audio recordings were manually transcribed and split into verb phrases, and two research assistants blind to the purposes of the study coded the verb phrases independently into facts, interpretations, and conversation regulation (Butner et al., 2008; Pasupathi and Hoyt, 2009; Pasupathi and Wainryb, 2010). Using the coding scheme, Facts are verb phrases relating to something present in the world and the five senses (e.g., “I see your red blocks”); Interpretations are verb phrases relating to “mental processes” and refer to inferences, emotions, or evaluations (e.g., “I think an airplane would be faster than a helicopter”); and Conversation regulations are verb phrases relating to backchanneling or establishing understanding (e.g., “I agree”). Agreement between coders was acceptable, *κ* = 0.78 for Minecraft, *κ* = 0.84 for NEO.

For each team’s coded transcript, inter-event codes (IECs) were calculated as the number of other codes between each occurrence of the code being analyzed (e.g., if interpretations are being analyzed, then the number of facts and conversation regulations between interpretation coded communication events comprise the IECs). The more dynamic organization a code has, the greater its role in team dynamics (Dunbar and Gorman, 2014).

To ensure that it was proper to use a power-law scaling exponent to measure dynamic structuring, we calculated the goodness-of-fit between the data and the power-law distribution using the methods recommended by Clauset et al. (2009). Their approach compares how well the empirical data fit a power-law distribution relative to how well samples of synthetic data fit a power-law distribution using the Kolmogorov–Smirnov statistic. We also compared the power-law distribution to alternative distributions including exponential, Poisson, and log-normal using a likelihood ratio test.

Assuming a significantly better power-law fit, the more negative the scaling exponent, the more emergent, or dynamically structured, the team communication process. This interpretation is consistent with Butner et al. (2008), Brown and Liebovitch (2010), and Gorman and Crites (2015). Relevant to the current study, we predicted significantly more dynamic structuring for interpretation-based communication for the NEO task and significantly more dynamic structuring for fact-based communication for the Minecraft task (Hypothesis 1).

#### Communication Flow

Communication flow was used as a measure of communication patterns to test Hypothesis 4. After each audio recording was transcribed, we used the program Audacity (Audacity, 2016) to calculate the start and end time of each utterance in the audio recordings using the sound finder function, which calculates the timestamps for any noises above a set decibel threshold (in this case, the Audacity default of 20 dB was used). The timestamps were then manually matched with the utterances in the transcript. Timestamps that did not match the utterances (e.g., a participant sneezing) were discarded. If there were utterances that did not have any matching timestamps, timestamps were obtained manually by measuring the start and ending points of the speech waveform in Audacity. The timestamps for each utterance were used to create a 1 Hz symbolic time series of who was talking for each second of the transcript. The six possible symbol states for the communication flow time series are shown in Table 2.

**Table 2:** Symbolic states for communication flow.

Code	Speaker
0	No speakers
1	Participant 1
2	Participant 2
3	Experimenter 1
4	Experimenter 2
5	Multiple speakers

To match the neural measures described in the next section, we calculated quantitative estimates of information transmission, or bits, on the symbolic communication flow time series using Shannon entropy (Shannon and Weaver, 1949).

Entropy is first measured, where *p_i_* is the relative frequency of distribution *i* over a sliding window (100 s). Entropy is first measured over the initial 100 s communication distribution, then the window is shifted forward 1 s, creating a new distribution by adding a symbol and deleting the first symbol until the window has been slid over the entire communication flow time series. For a symbolic time series of length *N*, this results in a continuously fluctuating entropy time series of length *N*-99. We used 100 s as the window size because prior research has indicated that windows smaller than 100 s can create artifactual spikes in the entropy time series (Likens et al., 2014).

$$Entropy=-\;\sum_{i=1}^{\#Symbol\;States}\left(p_{i}\times \log_{2}p_{i}\right)$$

#### Neural Measures

To obtain the neural activity from each team member, we used Cognionics Quick-20 Dry EEG headsets. These headsets included electroencephalography and a full 10-20 EEG array (20 channels plus mastoid reference and ground) which recorded the participants’ EEGs continuously throughout baseline measurement and task performance. The continuous EEG data were subjected to band-pass filtering at 0.1 (high-pass) to 30 (low-pass) Hz and 50 Hz notch filtered to isolate the electrical recording from possible environmental contamination using the open-source Matlab Toolbox EEGLab. Following the band-pass and notch filtering, blinks, and electromyography artifacts were removed in EEGLab using independent components analysis (ICA; Onton et al., 2006). ICA is used to create components that are maximally independent of one another, minimizing mutual information between the components. ICA was used here to remove the components associated with eye blinks and electromyography artifacts from the original EEG channel data, which were usually the first two components in the ICA corresponding to the Fp1 and Fp2 components. Finally, fast-Fourier transforms were conducted using EEGLab to split the neural data into the alpha and beta frequency bands.

**Figure 3:**
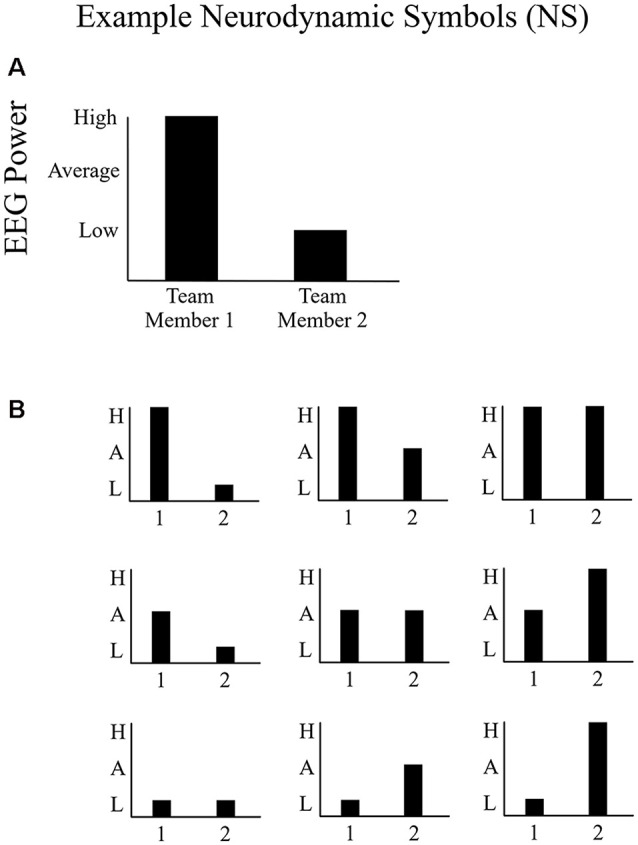
Example of neurodynamic symbols (NS). **(A)** An example NS representing above-average EEG power for one team member and below-average EEG power for the other team member and **(B)** the symbol space (NS states) of potential distributions of EEG power at any sensor location for any frequency between two people. The EEG power distribution could either be higher than baseline, around the baseline, or lower than baseline.

Each team’s neurodynamics was assessed each second through the relative levels of EEG power (amplitude^2^) for each team member. For all EEG channels and frequencies, the distribution of activation was sampled at 500 Hz to capture changes in the distribution (Figure 3A) over time. A set of neurodynamic symbols were created that classifies the activation distribution across a team as a discrete neurodynamic state (NS; Figure 3B; Stevens and Galloway, 2014, 2016). A set of nine neurodynamic symbols were created that show the activity levels for both of the team members individually as well as in the context of the other team member (Figure 3B; Stevens and Galloway, 2014, 2016). The activity levels are a split of the upper 33% (high), middle 33% (average), and lower 33% (low) EEG power levels relative to the average baseline power. The average baseline power was calculated from the 15-min baseline for each participant, described under “Participants and Procedure” section, captured before task performance. For each second, the EEG power levels for each participant during the task session was compared to their individual average baseline EEG power level and subsequently categorized into the high (higher than average baseline), average (around average baseline), and low (lower than average baseline) categories for each participant using the 33% cut-offs described earlier. These individual symbolic series were then combined into a team-level symbol series (NS 1–9 in Figure 3) that shows the activity levels for both team members individually but also in the context of the other team member at each second. An NS symbol series, sampled at 1 Hz, is the result and provides the input for the neurophysiological synchronization analysis. This process was conducted separately for the alpha and beta frequency bands.

Often, synchronization is characterized as 1:1 phase locking between two signals, perhaps because it is frequently described this way in the psychological literature (e.g., mirroring; Sternad et al., 1992). However, synchronization is more generally defined as frequency locking between two signals in any proportion (e.g., 2:1; 3:1; Gorman et al., 2017a). To quantify neurophysiological synchronization according to this more general definition of synchronization, we calculated synchronization across all amplitude combinations (NS) shown in Figure 3. The assumption is that teams synchronize by either both members being low or being high (i.e., mirroring) or by one team member is high and the other low (non-mirroring synchronization), and so on, so long as NS relationship is relatively stable over time.

Neural synchronization was operationally defined as the relative stability (vs. variety) of symbols in the NS symbol series over time as indexed by Shannon information. Shannon entropy was calculated on the neurophysiological symbolic time series to obtain a quantitative estimate of NS distribution variety over time. For example, the greater the variety of the symbol distribution in a 100 s window, the higher the entropy and the lower the synchronization. Sliding window Shannon entropy was used to provide a continuous measure of synchronization and to match the sliding window measurement of communication flow entropy. Decreased entropy can be operationally defined as higher synchronization because the NS distribution is not changing (is stable) over time, whereas higher entropy can be operationally defined as lower synchronization because the NS distribution is changing over time. We chose Shannon entropy as our measure of synchronization because other approaches such as cross-correlations or phase-phase coherence provide only linear correlations between signals over time (Pereda et al., 2005), whereas our approach provides a continuous measure of synchronization beyond 1:1 phase locking to capture all modes of synchronization (linear and nonlinear) that has been established to neural synchronization in complex team tasks (Stevens and Galloway, 2014, 2016). Additionally, the EEG time series power spectra from the current study exhibited non-stationarity, to which linear synchronization approaches are less sensitive (Pereda et al., 2001).

Entropy serves as the measure of synchronization in neural activity to address Hypotheses 2, 3, and 4. There are two entropy time series for each team, one for Interaction A (team member communication) and one for Interaction B (experimenter communication). For Hypothesis 2, synchronization across participants is expected to only occur when team members are working on the task together and not when working with an experimenter. For Hypothesis 3, synchronization was examined in the context of specific sensor sites depending on task type. The NEO task should affect the mu medial and phi complex rhythms in the alpha band (8–12 Hz) in the Fz, POz, and P4 electrodes, and the Minecraft task should affect the mu and low beta rhythms in the beta band (13–30 Hz) in the Fz, C3, CZ, and C4 electrodes. For Hypothesis 4, synchronization was examined across neurodynamics and communication flow using lagged cross-correlations. We expected to find significant cross-correlations between communication flow and neural synchronization as evidence of cross-level effects in teams.

#### Subjective Measures

Participants completed a survey at the end of the experimental session containing questions about their demographics (e.g., gender, age, year in college, major, race and ethnicity), factors that could confound their task performance (e.g., hours spent playing video games per week, hours spent playing social games per week, familiarity with the game Minecraft, prior military experience), factors that could confound their EEG data (e.g., caffeine, nicotine, or alcohol consumption; neurological or psychiatric disorders), and how frequently they move their hands and head while speaking as indicated on a five-point Likert frequency scale. The variables from this survey were used as covariates for some of the analyses described in the “Results” section.

## Results

### Effect of Task Type on Communication Structure

To test the hypothesis that team communication is dynamically structured based on the type of task, a preliminary analysis was conducted comparing the fit of the empirical data to synthetic datasets using the Kolmogorov–Smirnov statistic. This comparison was conducted for all 34 of the task sessions and the three types of coded communication: Facts, Interpretations, and Conversation Regulation. According to recommendations by Clauset et al. (2009), if the *p*-value is greater than 0.1, then this suggests that a power-law is a plausible distribution for the dataset. We also ran a binomial test on the proportions of the task sessions where a power-law distribution seemed to be a plausible fit to determine if the proportion was statistically significant. The results indicated that for Facts, power-law was a plausible distribution for 21 of the 34 task sessions which were not a significant proportion of the data, *p* = 0.05. For Interpretations, power-law was a plausible distribution for 23 task sessions which was statistically significant, *p* = 0.02. For Conversation Regulation, power-law was a plausible distribution for 25 task sessions which was also statistically significant, *p* = 0.003. These findings suggest that a power-law distribution is plausible for Interpretation and Conversation Regulation communication, but not for Facts.

**Figure 4:**
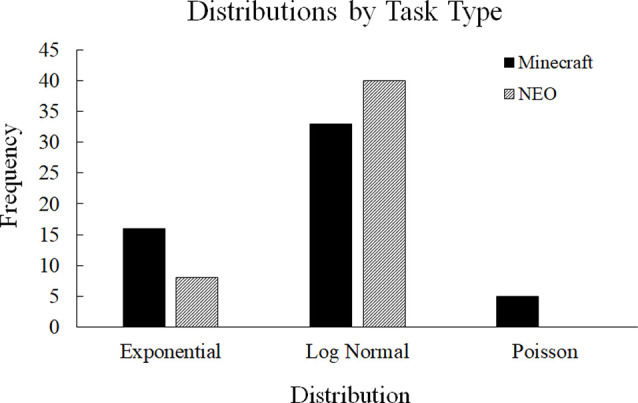
Distribution differences based on the type of task. The Minecraft and NEO tasks differed primarily in how many datasets were the best fit by an exponential distribution and a Poisson distribution.

To rule out alternative distributions, we compared the power-law distribution to alternative distributions that look like a power-law (exponential, Poisson, and log-normal) using a likelihood ratio test (Clauset et al., 2009). We determined which type of distribution best fit each dataset based on the likelihood ratio test results, which revealed that none of the datasets was the best fit by a power-law. However, the datasets differed on whether they were the best fit by exponential, Poisson, and log-normal distributions. We used a chi-square test of independence to determine if there were significant differences in these distributions based on either the Task Type (Minecraft or NEO) or the Communication Code (Facts, Interpretations, or Conversation Regulation). The Task Type by Distribution chi-square was significant, $\chi_{\left(2\right)}^{2}$ = 8.01, *p* = 0.02, suggesting that the two task types resulted in different communication distributions, which provides some support for Hypothesis 1. Based on Figure 4, the two tasks primarily differed in the number of datasets that were best fit by an exponential distribution and a Poisson distribution. However, the Communication Code by Distribution chi-square analysis was not significant, $\chi_{\left(4\right)}^{2}$ = 8.79, *p* = 0.07, suggesting that the distributions did not differ based on the type of communication code. These latter results do not support the hypothesis that team communication is dynamically structured around specific communication types (i.e., Facts vs. Interpretations) based on task type; however, the former result suggests that task type did manipulate the underlying communication distribution independent of code type.

### Effect of Interaction on Neural Synchronization

To test the effect of the Interaction condition on neural synchronization, we conducted separate 2 (Task Type) × 2 (Interaction) × 2 (Trial Order) mixed ANOVAs for the electrodes Fz, P4, and POz in the alpha band as well as Fz, Cz, C3, and C4 in the beta band. Because these were planned comparisons, no alpha correction was used. We also conducted exploratory *post hoc* contrasts with a Bonferroni alpha correction (*α* = 0.008) in the alpha and beta bands in the frontal, central, and parietal cortical regions. These three regions were selected because they represent the broader regions for the individual electrodes tested in Hypothesis 2 and Hypothesis 3.

Based on the second hypothesis, we predicted that neural synchronization with a team member will only occur when talking to a team member and not when talking to an experimenter. However, no main effects of Interaction were statistically significant. Controlling for the amount of communication (both how much time was spent speaking and the number of utterances produced during the session) as well as the self-reported frequency of hand and head movements when speaking did not alter the pattern of results. Additionally, the pattern of mean differences was not in the hypothesized direction: Means for the Experimenter condition were lower (indicating higher synchronization) than in the Participant condition across both task types for all electrodes, regions, and frequencies tested. Both sets of results fail to support Hypothesis 2.

To ensure that any observed effects were not due to the order of the trials participants engaged in, we conducted similarly planned and exploratory comparisons investigating the effect of Trial Order on neural synchronization. The between-subject comparisons of AB and BA, where A refers to the Team Member condition and B refers to the Experimenter condition, were tested. As expected, the pattern of results was null (all *p* > 0.05), indicating that differential transfer did not occur when going from A to B vs. going from B to A.

### Effect of Task Type on Neural Synchronization

We used the same mixed ANOVAs from Hypothesis 2 to test Hypothesis 3. For the third hypothesis, we predicted that the pattern of neural synchronization between team members would depend on the type of team task. Specifically, we predicted increased neural synchronization in the alpha band in the Fz, POz, and P4 electrodes for the NEO task and increased neural synchronization in the beta band in the Fz, Cz, C3, and C4 electrodes for the Minecraft task. This hypothesis was only partially supported for the NEO task in the Fz electrode, where the NEO condition’s (*M* = 2.53, *SD* = 0.08) neural entropy in the alpha band was significantly lower than in the Minecraft condition (*M* = 2.99, *SD* = 0.08), *p* = 0.002. There was a similar result in the Fz electrode in the beta band, but the results were in the opposite direction from what was predicted: the NEO condition’s (*M* = 2.70, *SD* = 0.09) neural entropy in the beta band was significantly lower than in the Minecraft condition (*M* = 3.08, *SD* = 0.09), *p* = 0.02. No other main effects of Task Type were significant. Controlling for the amount of communication (both how much time was spent speaking and the number of utterances produced during the session) as well as the self-reported frequency of hand and head movements when speaking did not alter the pattern of results.

Additional analyses focusing on the 2 (Task Type) × 2 (Interaction) component of the 2 × 2 × 2 mixed ANOVA with the same electrodes, frequency bands, and brain regions suggested that the effect of Task Type did not depend on the Interaction condition.

### Cross-level Effects

**Figure 5:**
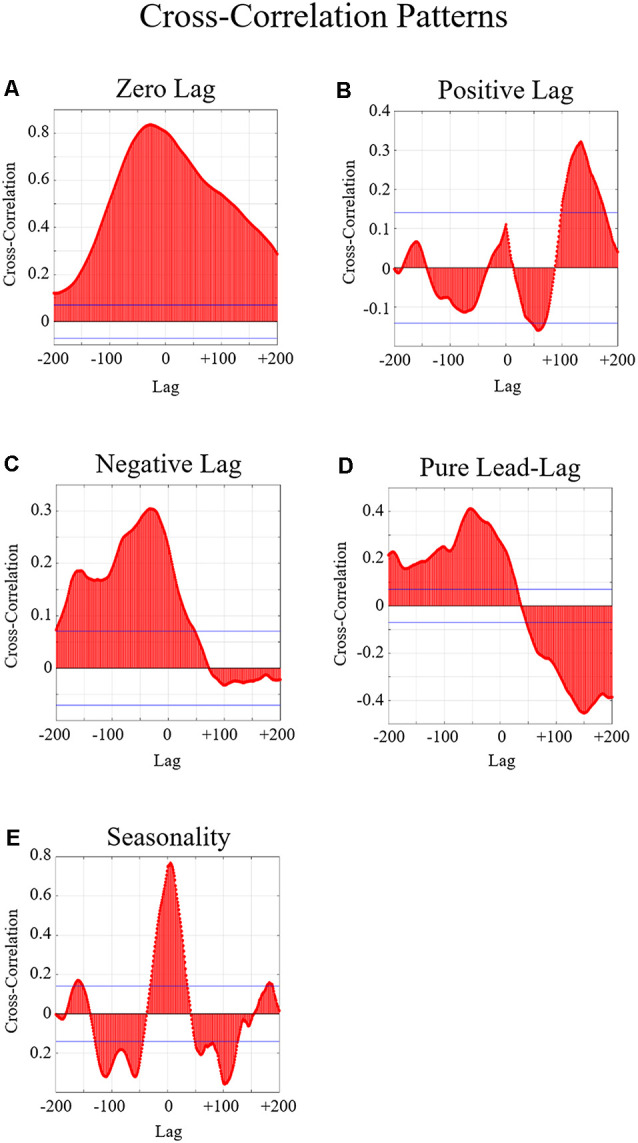
Examples of the five possible relationships examined in the cross-correlation graphs. In these graphs, the *x*-axis indicates the time lag where 1 lag = 1 s. The *y*-axis indicates the cross-correlation between the neural entropy and the communication flow entropy. The blue horizontal lines indicate the threshold of significance. Any peaks beyond this line are considered statistically significant correlations. **(A)** An example of a significant zero-lag relationship is indicated by a significant positive or negative peak around lag zero. Significant zero-lag relationships indicate that the two variables change together. **(B)** Example of a significant positive lag relationship which is indicated by a significant peak in positive lag. **(C)** An example of a significant negative lag relationship is indicated by a significant peak in negative lag. Significant positive and negative lags indicate that changes in one variable precede the other variable. **(D)** An example of a significant pure lead-lag relationship is indicated by a single significant positive peak and a single significant negative peak in both forward and backward lag. Significant pure lead-lag relationships indicate that changes across both variables alternate which one precedes or follows the other. **(E)** Example of a significant pure lead-lag with seasonality relationship which is indicated by multiple significant positive and negative peaks in both forward and backward lag. Significant pure lead-lag with seasonality relationships indicate that changes in the two variables occur across multiple points in time.

The fourth hypothesis was that communication and neural activity should be related, such that changes in communication patterns should be reflected in the neural activity of teams (and vice versa). To investigate this hypothesis, we calculated lagged cross-correlations using the MatLab crosscorr function for each team across 17 electrodes (C3, C4, Cz, F3, F4, F7, F8, Fz, O1, O2, P3, P4, P7, P8, Pz, T3, and T4), two frequency bands (alpha and beta), and both Interaction conditions (experimenter and team member). Similar to prior research (Gorman et al., 2016), we examined the graphs of the lagged cross-correlations for qualitative relationships of significant zero-lag (i.e., significant positive or negative peak at lag 0; changes in the communication flow and the neural activity occur simultaneously), positive lag (i.e., significant peak in positive lag; changes in the communication flow precede changes in the neural activity), negative lag (i.e., significant peak in negative lag; changes in the neural activity precede changes in the communication flow), pure lead-lag (i.e., a single significant positive peak and a single significant negative peak in both forward and backward lag; changes in the neural activity alternate between preceding or following changes in the communication flow), or pure lead-lag with seasonality (i.e., multiple significant positive and negative peaks in both forward and backward lag; the neural activity and communication flow covary regularly across multiple points in time). See Figure 5 for examples of these patterns. The lag in each graph corresponds to 1 s in time. To analyze the cross-correlations for significant differences in pattern types, we conducted chi-square tests of independence to determine how the Interaction, Task Type, or Trial Order manipulations affected the pattern of temporal cross-correlations.

#### Experimenter vs. Participant Conditions

**Figure 6:**
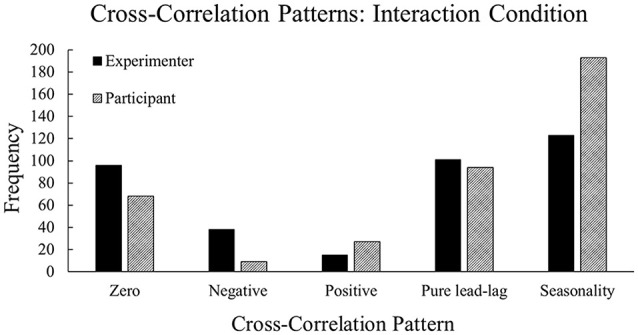
Frequencies of cross-correlation patterns observed across the experimenter and participant conditions. The experimenter condition contains higher frequencies of zero-lag and negative lag whereas the participant condition contains higher frequencies of seasonality and positive lag.

There were significant differences in the type of patterns observed between the neural entropy and the communication flow entropy time series depending on who the participant was performing the task with (Figure 6), $\chi_{\left(10\right)}^{2}$ = 822.33, *p* < 0.001. In the experimenter condition, we observed more instances of zero-lag and negative lag compared to the participant condition. In the participant condition, we observed more instances of seasonality and positive lag compared to the experimenter’s condition. These patterns suggest that the temporal relationship between team neural entropy and communication flow was affected by the Interaction condition. Specifically, the experimenter condition produced a more transient relationship across time (i.e., neural entropy and communication flow entropy change together at the same time), whereas the participant condition produced a more persistent relationship across time (i.e., neural entropy and communication flow entropy are related across multiple points in time). These results support Hypothesis 4.

#### Minecraft vs. NEO

**Figure 7:**
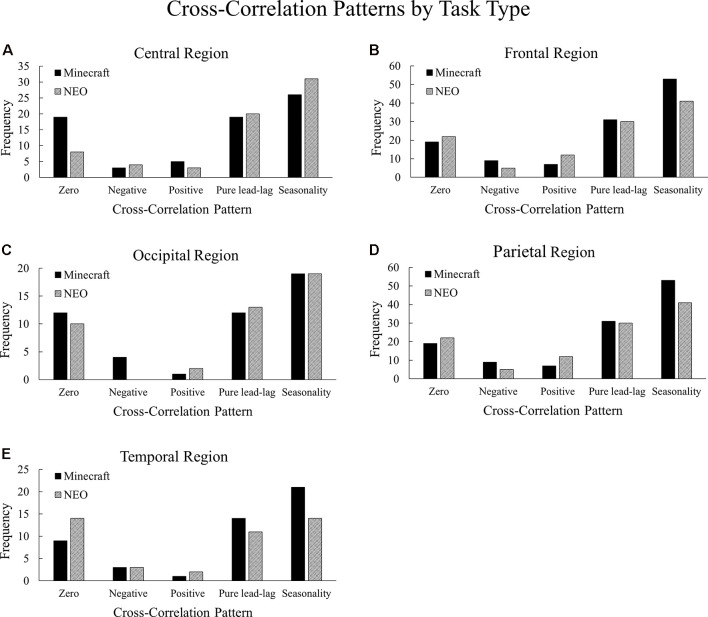
Frequencies of cross-correlation patterns observed across the Minecraft and NEO task conditions, with **(A)** central, **(B)** frontal, **(C)** occipital, **(D)** parietal, and **(E)** temporal regions. The Minecraft condition contains higher frequencies of zero-lag in the central region, seasonality in the frontal and temporal regions, and negative lag in the occipital and parietal regions compared to the NEO condition.

The cross-correlation patterns between the Minecraft and NEO task conditions were compared within the central, frontal, occipital, parietal, and temporal brain regions collapsing across the alpha and beta frequencies because we did not observe significant differences in the patterns between the alpha and beta frequency ranges, $\chi_{\left(4\right)}^{2}$ = 2.01, *p* = 0.73. All Task Type comparisons were statistically significant: central, $\chi_{\left(10\right)}^{2}$ = 810.17, *p* < 0.001; frontal, $\chi_{\left(10\right)}^{2}$ = 793.21, *p* < 0.001; occipital, $\chi_{\left(10\right)}^{2}$ = 817.22, *p* < 0.001; parietal, $\chi_{\left(10\right)}^{2}$ = 794.01, *p* < 0.001; and temporal, $\chi_{\left(10\right)}^{2}$ = 805.54, *p* < 0.001. See Figure 7 for a visualization of these differences.

In the central region, the Minecraft task had more instances of zero-lag relationships compared to the NEO task, suggesting that for the Minecraft task in the central region, neural entropy and communication flow entropy changed together at the same time more than the NEO task. In the frontal region, the Minecraft task had more instances of seasonality relationships and less positive lags compared to the NEO task, suggesting that for the Minecraft task in the frontal region, neural entropy and communication flow entropy were related to one another across multiple points in time. The two tasks differed slightly in the occipital region where the Minecraft task had more instances of negative lag than the NEO task, suggesting that changes in the neural entropy led to changes in communication. For the parietal region, the Minecraft task again had more instances of negative lag whereas the NEO task had more instances of positive lag, where positive lag indicates that communication changes led to neural changes. Lastly, in the temporal region, the Minecraft task contained more seasonality patterns compared to the NEO task whereas the NEO task contained more zero-lag patterns.

The results for task differences in the cross-correlations suggest that the relationship between neural entropy and communication flow differs across the brain regions depending on the type of task. For the Minecraft task, the frontal and temporal regions indicated long-term dependencies between neural synchronization and communication flow, whereas the central region indicated that the neural synchronization and communication flow changed together only “at the moment.” For the NEO task, the opposite pattern was observed—the central region indicated long-term dependencies whereas the frontal and temporal regions indicated immediate changes. This set of findings provides support for Hypothesis 4.

#### First vs. Second Trial

**Figure 8:**
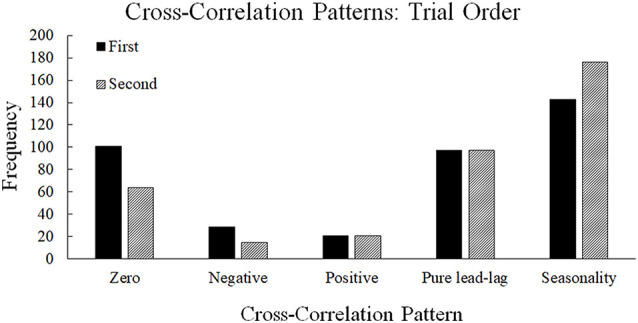
Frequencies of cross-correlation patterns observed across the first and second trials. The first trial contains higher frequencies of zero-lag and negative lag whereas the second trial contains higher frequencies of seasonality.

There were significant differences in the type of patterns observed between the neural entropy and the communication flow entropy time series depending on the trial order (Figure 8), $\chi_{\left(10\right)}^{2}$ = 796.08, *p* < 0.001. In the first trial, there were more zero-lag and negative lag relationships observed between the neural entropy and the flow entropy time series (Figure 8). This pattern indicates that the neural entropy and communication flow entropy changed together at the same time in a more transient fashion during the first trial. By the second trial, the relationship between the neural and flow entropy time series more frequently showed a pure lead-lag with seasonality relationship. This pattern suggests that neural entropy and communication flow entropy were related to one another across multiple points in time in the second trial. Both sets of results suggest that there may have been transfer (order effects) across trials in the relationship between neural entropy and communication flow entropy, such that long-range dependencies (persistence) developed between neural synchronization and communication flow from the first trial to the second trial (see also Gorman et al., 2016, for similar results regarding less- vs. more-experienced teams).

## Discussion

This study demonstrates how specific team task constraints can differently structure team communication and neural dynamics over time. Specifically, we found support for cross-level effects in the form of significant cross-correlations between changes in the neural synchronization of teams and changes in the team’s communication flow across time, which was affected by the type of task the team was performing and whether the team members were performing the task with each other. Although we were unable to replicate previous research findings where manipulating the type of task alters the dynamical structure of specific team communication codes, we did find differences in the communication patterns due to the type of task. However, we did not find support for the effect of task type and interaction partner on neural synchronization across the hypothesized electrodes, frequency bands, or brain regions. It appears that while we can control communication structure through team task constraints, it is more difficult to pinpoint concomitant team neural synchronization effects beyond the task and interaction partner-dependent patterns of temporal cross-correlation observed in this study (see also Gorman et al., 2016).

### Communication Dynamics

The first hypothesis was both theoretical in that communication dynamics should match task dynamics (e.g., action-based tasks should modulate action-based communication) and a manipulation check based on prior work (Dunbar and Gorman, 2014) using a shorter task period (15 min compared to the original 45 min). In Hypothesis 1, we predicted that the action-based Minecraft task would result in dynamical communication structuring around fact-based communication, and the planning-based NEO task would result in dynamical communication structuring around interpretation-based communication. Although task type resulted in significantly different communication pattern distributions, the dynamic structuring of task-specific communication codes hypothesis was not supported, and it is plausible that one of two things is occurring: That the dynamics in the communication data are illusory or that the 15-min time frame for the task was too short to produce reliable fractal, power-law dynamics. The latter interpretation may be plausible because time series shorter than 256 data points can produce inaccurate estimates of the time series’ true scaling exponent (Delignieres et al., 2006). Dyads varied in the amount that they communicated with one another. On average, dyads spent less than half the task session communicating [time (s) spent speaking *M* = 419.00, *SD* = 189.75]. The average length of verb phrases (verb phrase *M* = 259.97, *SD* = 104.54) for each task session is near the point where the fractal analysis may not accurately capture the scaling exponent, potentially impacting the reliability of the scaling exponents for the teams that communicated less frequently. Future research is necessary to determine the optimal task length to circumvent this issue for the scaling exponents.

### Neural Synchronization

The second and third hypotheses pertained to the effect of the Interaction (H2) and Task Type (H3) conditions on the average neural entropy. In Hypothesis 2, we predicted that neural synchronization would be higher in the team member condition compared to the experimenter condition. In Hypothesis 3, we predicted increased neural synchronization in the alpha band in the Fz, POz, and P4 electrodes for the NEO task and increased neural synchronization in the beta band in the Fz, Cz, C3, and C4 electrodes for the Minecraft task. Neither hypothesis was supported by the results of this study. Rather, we found that the Fz electrode identified differences in neural synchronization between the NEO task and the Minecraft task, similar to an indicator found in prior research (Stevens and Galloway, 2014, 2016, 2017; Gorman et al., 2016).

One possible reason for the null results may be because we averaged entropy across the entire task session, masking changes in entropy in the time series due to a loss of the internal data structure. By averaging across the task session, we may have removed nuances in the neural entropy over time that could have been due to the task type or interaction conditions. This argument has some support based on the differences between the averaged results and the time series results, which are discussed in the next section “Cross-level Effects.”

Another possible reason for the null results may be because the teams were coordinating during “typical conditions.” Other research has used neural measures of entropy as an indicator of uncertainty, where high entropy represents high uncertainty or “surprise” that needs to be resolved by the team (Stevens and Galloway, 2017). Prior research has found that teams tend to have low-to-average neural entropy as they work in typical conditions and that entropy increases with uncertainty and decreases after uncertainty reach a tipping point, or threshold, where the team needs to resolve the uncertainty during the task session (e.g., Stevens et al., 2016). Uncertainty can be inherent to the task setting, task performance, or introduced artificially in the task session by perturbing the team during task performance. We did not include perturbations in this study, which may have resulted in a lack of variability in neural entropy. Also, both the NEO and Minecraft tasks have less uncertainty, or surprise, built into them than more realistic tasks, such as medical simulations (e.g., Stevens et al., 2016). It may also be plausible that the relationship between entropy and uncertainty is mediated by attention, where entropy is an indicator of when joint attention is needed from the team to resolve uncertainty in the task. The tasks selected for this study had few incidents where participants would need to jointly attend to an event on their screen.

It is also possible that neither the task type nor interaction manipulation affected neural dynamics. Although they modulate the distribution of the communication content, the task type manipulations might not affect neural synchronization similarly across the brain. In the average neural entropy results, we found only that the Fz electrode identified reliable differences in neural entropy between the Minecraft and NEO tasks. The cross-correlation analyses indicated that there were differences due to task type when examining neural entropy over time (rather than averaging); however, there was a fair amount of overlap between the cortical regions involved in the relationship between the neural activity and communication across the two task types. These results provide only limited support for extending the theory of interactive team cognition (Cooke et al., 2013) to neural coordination and point to the current limitation in specifying neural differences at the level of detail required to test scalp location- and frequency-based differences using these types of team tasks.

The interaction manipulation was based on prior research on interpersonal motor coordination dynamics; namely, postural sway (Shockley et al., 2003). In prior studies, this interaction manipulation served as a convenient control to disentangle dynamics due to the experimental task from dynamics due to the shared interaction medium (i.e., verbal coupling). In the average neural entropy results, we did not find any differences in direct verbal coupling between the participants. The null results for the interaction condition may be because we overestimated the impact that the experimenter and participant task sessions would have on the verbal coupling. Specifically, whether the participants were communicating with each other or communicating with the experimenter, they were still performing the same task, such that residual neural synchronization would occur regardless of the interaction partner. Although the Interaction condition may not have successfully manipulated neural dynamics on its own, it was successful in modulating the cross-level effects between neural synchronization and communication flow.

### Cross-level Effects

In the fourth hypothesis, we expected to find reciprocal relationships between the communication and neural activity of teams based on predictions from the dynamical systems theory of team coordination (Gorman, 2014; Gorman et al., 2017b, 2020). We addressed this hypothesis using lagged cross-correlation analysis. We found support for this hypothesis, where significant changes in communication flow were temporally cross-correlated with the neural entropy time series of teams. These results provide support for the dynamical systems theory of team coordination by demonstrating different patterns in cross-level effects as a function of changing team task constraints.

Unlike the average neural entropy results, we found that the relationship of neural entropy with communication flow was impacted by the Interaction condition. Specifically, we found significant zero-lag and negative lag relationships between neural entropy and communication flow entropy in the experimenter condition, whereas we found significant pure lead-lag with seasonality and positive lag relationships in the participant condition. The results for the experimenter condition suggest that the relationship between changes in neural activity and who is speaking and when is more superficial because they are only related momentarily in time. If this relationship were meaningful (e.g., tied to the speaker), there would be significant relationships across time. Conversely, we expected to find significant relationships across time for the participant condition because, in this condition, the communication flow was relevant not only to what was going on in the task now, but also previously, and in the future for *both* participants. Conversely, for the experimenter condition, communication flow was only relevant for one participant at a time, because the participants were performing the task separately with different experimenters. Also, we found that the relationship between neural entropy and communication flow was affected by the Task Type condition. We found more pure lead-lag with seasonality patterns primarily in the frontal and temporal regions for the Minecraft task. For the NEO task, we found more pure lead-lag with seasonality patterns in the central region. Taken together, these findings suggest that the communication—neural activity link in teams as a function of task type and interaction partner only becomes apparent when the relationship is examined as a pattern extended in time rather than as a mean over time as in the aggregate communication distribution and neural synchronization analyses.

Although we performed these analyses to examine Hypothesis 4, the differences found in the relationship between the communication and neural activity of teams were not at all similar to the predictions in Hypothesis 3. In contrast to Hypothesis 3, it seems that multiple cortical regions contribute to the relationship between neural activity and communication flow of teams, which overlap somewhat between the two task types. The predictions for Hypothesis 3 were derived from prior research investigating the neural coordination between interacting individuals performing different types of tasks, but much of this research focused primarily on the frontal (e.g., Stevens and Galloway, 2014, 2016, 2017) and central regions (e.g., Moreno et al., 2013; Stevens and Galloway, 2014, 2016), with some emphasis on the parietal-occipital region (e.g., Stevens and Galloway, 2016, 2017). In contrast, similar to the results found in the current study, basic speaker-listener coupling research suggests that neural coupling occurs widely across the brain, relative to both language production and comprehension (Stephens et al., 2010). With communication flow, we found that the temporal region seems to contribute most to the relationship between neural activity and communication flow, which is compatible with findings from speaker-listener coupling in the temporal regions concerning language production (Stephens et al., 2010). Our findings suggest that in addition to the frontal and central regions, future research on team coordination dynamics should measure the temporal region in conjunction with team communication to determine how these cortical regions are differentially related to team communication.

### Limitations and Future Directions

Although, we controlled for how much participants and experimenters communicated and participants self-reported movement during speech in our analyses, we did not control for experimenter experience with the task nor task performance. Prior research has shown that task experience affects the relationship between neural activity and communication (Gorman et al., 2016). The trained experimenters were quite experienced with the task and ran their task sessions similarly across sessions, whereas the participant sessions differed in how much the participants communicated and how the participants approached the task. Also, we were unable to measure task performance due to the infeasibility of acquiring accurate performance measures, but the two sessions may have differed on their task performance. It may be possible that the experimenter’s expertise with the task influenced the participant’s neural and communication dynamics in unintended ways. Future research will be necessary to disentangle this by recruiting four naïve participants for each experimental session, rather than having two participants work separately with trained experimenters.

Another limitation of this study was that it was difficult to find changes in neural entropy in the Minecraft task. The Minecraft task is a predictable and relatively unchanging task with little uncertainty. Prior research usually inserts identifiable events meant to disrupt the team’s coordination to induce changes in dynamical parameters in the time series (e.g., Gorman et al., 2016). Future research should perturb the team as the team members are coordinating to induce variability in neural dynamics or use tasks with more built-in uncertainty (e.g., during healthcare simulation training; Stevens et al., 2016).

Another possible future direction is to examine other approaches for conducting the neural analysis. Individual participants’ neural data were discretized into a team level time series by averaging into one-second epochs and converting the two team member’s neural data into NSs before running the entropy analysis. Although widely used in team neurodynamics research, this discretization process could have obscured some information in the continuous amplitude time series relevant to neural synchronization. Prior research has suggested that neural synchronization occurs at a delay of a few seconds (Stephens et al., 2010; Kuhlen et al., 2012), however, so it may also be possible that the discretization process did not obscure relevant information. Future research is needed that catalogs the qualities of and differences between varieties of neural synchronization analyses for investigating neural synchronization in teams.

## Conclusion

The purpose of the current study was to establish whether cross-level effects exist in teams between the communication and neurophysiological levels of analysis and whether they can be controlled by constraining how teams coordinate. The current findings illustrate that teams do dynamically structure the flow of their communication in relation to their neural activity. This dynamical structuring across multiple levels of analysis occurs around both the task constraints and who the team is working with on the task, but only when looking at the time series; these relationships may be concealed when looking for cross-level effects using aggregated neural and communication measures. These findings highlight the need to examine the nuances of the relationship between levels of analysis—cross-level effects—across time. Ultimately, investigating the causal nature of these relationships may reveal how neural and communication levels of analysis are nested within one another, providing new conceptualizations of team process measurement for assessing team performance over time.

## Data Availability Statement

The datasets generated for this study are available on request to the corresponding author.

## Ethics Statement

The studies involving human participants were reviewed and approved by Georgia Institute of Technology Institutional Review Board. The participants provided their written informed consent to participate in this study.

## Author Contributions

TD conceptualized and conducted the research and primarily wrote the manuscript. JG contributed to the conceptualization of the research and revising and editing the manuscript.

## Conflict of Interest

The authors declare that the research was conducted in the absence of any commercial or financial relationships that could be construed as a potential conflict of interest.
